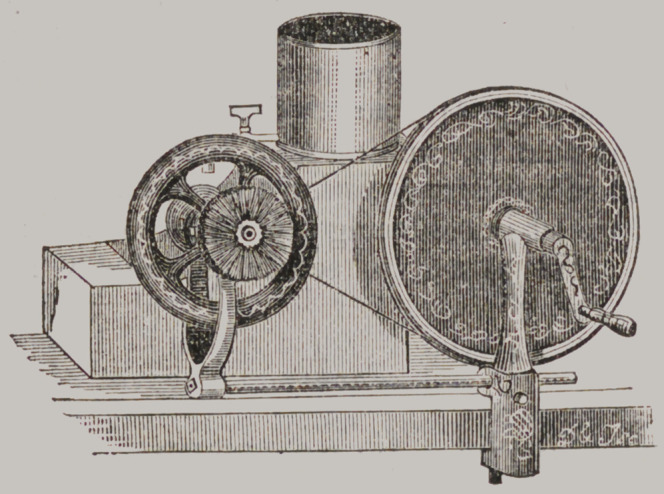# Cuttings, Obituary, Notices, &c.

**Published:** 1858-11

**Authors:** 


### ARTIFICIAL STONE.

A new combination of mineral substances for the production of artificial stone, has been provisionally specified by Mr. F. Puls, of Haverstock-hill. The invention has reference to the production of artificial stone for ornamental and other purposes, and consists in the combination of powdered emery, flint-glass, ruby, diamond, melted alumina, oxide of iron, or similar hard mineral substances, with proportionate quantities of lime, barytes, plaster of paris, or chalk, and silicate of potash or soda, or potash and soda powdered in solution, or in a semi-fluid state. For the production of stone for lithographic or ornamental purposes, he combines lime or chalk powder with silicate of potash or soda, or otherwise, to which coloring matter may be added as required; and for meerschaum, mixes carbonate of magnesia or oxide of magnesia, or a mixture of both, with silicate of potash, soda, or otherwise, to which may be added small proportions of slaked lime, chalk, or clay. Either of the above compositions may be pressed into molds, warm or cold, to give it the required shape, and render it close and compact.—*The London Builder.*

### ARTIFICIAL IVORY.

A patent has recently been granted in England to Charles Westendarp, Jr., for manufacturing a material which is made to imitate ivory, bone, horn, coral, or other similar substances, natural or artificial, and which may be used in preference to ivory, on account of cheapness and adaptability, as the same materials may be moulded or turned to the various forms or patterns they may be desired to take, and may be applied to all the purposes in which natural ivory becomes useful, such, for instance, as billiard-balls, door and other knobs, pianoforte keys, rulers, paper knives, whip, stick, and other mounts, and in imitation, or as a substitute for carved wood, enameled china, precious stone works, and a variety of fancy, ornamental, and decorative figures.

The process being as follows: Five ounces (or more or less according to the size of the article to be made), of ivory dust is soaked with a white color, say white lead or zinc white, three ounces, in a solution of white shellac or copal, in sixteen ounces of spirit of wine. After the whole is well mixed, which is best done at a temperature of 212° Fah., the alcohol is partially evaporated, and the stiff paste or dry powder pressed into a solid mass in the dies or mold, which have been previously heated to about 230° or 280° Fah.; after being so solidified they are polished in the ordinary manner of polishing ivory. Instead of using ivory dust, steamed and finely powdered bones, porcelain, cotton, and various finely powdered materials may be employed, and the colors may be varied according to the tint or shade required. If this invention will answer all the purposes of ivory, and can be made as perfect an imitation as the inventor claims, it will certainly be a very valuable acquisition to the trade, and will introduce very many beautiful ornaments into our parlors and elsewhere, besides many useful articles can be made which will cost comparatively a trifle to those wrought from the natural ivory.

### OREIDE—A NEW METAL.

By combining the undermentioned materials in the specified proportions, viz:—Pure copper, 100 parts in weight; zinc, 17 parts; magnesia, 6 parts; sal ammonia, 3·60 parts; quick lime, 1·80 parts; common tartar 9—Messrs. Mourier and Vallent have succeeded in forming an alloy closly resembling gold. This result was brought about by melting the copper in a crucible, and afterward adding by degrees, and in a pulverized form, the magnesia, sal ammonia, lime, and tartar. The whole is then briskly agitated for thirty-five minutes, and the crucible is then uncovered, the scum carefully removed, and the alloy poured into moulds, formed either of damp sand or metal. This alloy is fusible at a temperature which will readily admit of its being used for purposes of ornamentation; it possesses a fine grain, is denticulated, malleable, and susceptible of the most brilliant polish. When it has become tarnished by oxidation, its brilliancy is soon restored by means of a small amount of acidulated liquid. By simply substituting tin for zinc, a still more brilliant alloy is obtainable.

### NEW METALLIC ALLOYS.

In constructing apparatus for chemical purposes, philosophical apparatus, gold and silversmith's work, jewelry surgical instruments, alloys of either rhodium, iridium, or anthenium, or of several of these metals with platina, have not been used, but they have not hitherto been applied in fixed proportions to purposes of industry.

Henry Desmontis, of Paris, has obtained a patent in Great

Britain for the preparation and application of the alloys above mentioned, in any determined or definite proportions, to ornamental and useful purposes. The process is as follows: The metals in question are purified by the ordinary processes, and reduced to a pulverulent state; they are then mixed one by one, or several of them, with pulverulent platina, in certain determined and definite proportions (and such as are required for the respective purposes of industry to which the alloys are to be applied); the whole quantity being reduced to a minutely divided state and combined together, is then aggregated into masses or ingots in the usual way for platina itself, and solidified by heat as usually practiced in solidifying that metal. The object of the inventor is to furnish alloys in certain fixed quantities or proportions, according to the quality of the alloy, and the purpose for which it is required.

### THE DISCOVERY OF ELECTRICITY.

The casual rubbing of a little bit of amber produced the first recognized development of electricity, some six centuries before the Christian era; and this insignificant resinous deposit has thus given its Greek name to the wonderful agent whose greatest marvel we have just announced. For nearly 2,400 years, however, electricity was regarded as only an eccentric quality of amber and a few other articles; for, it is little more than a century since the discovery of the electric shock at Leyden, and during the last century all the grand triumphs of electricity have occurred.

### A WHOLESALE DENTAL OPERATION.

During a thunder shower that passed over the town of Petersham, Mass., last week, a bolt of lightning passed down a rod attached to the residence of a Mrs. Pierce. The lady was sitting at the window, immediately adjacent to the rod at the time, and was somewhat stunned. But she was greatly astonished to find, on examination, that every one of her upper teeth had been extracted by the shock without her knowing it, and were lying about loose on the floor.

Electricity moves with a greater velocity than light, which traverses 200,000 miles of space in a second of time.

### PLASTER IMPRESSIONS.

In taking plaster impressions for partial sets,—particularly when the remaining teeth are *irregular*,—much difficulty is experienced in removing the plaster from the mouth. Dr. S. Wardle, of this city, has exhibited to us an ingenious contrivance, of his own construction, which entirely obviates the difficulty. It consists of an ordinary impression cup, with a hole drilled through the center, and a V shaped plate about the size of a silver 25-cent piece, (different sizes are used, to correspond with the palatine arch), convex on one side, and concave on the other. About the center of the concave side, is soldered a small rod or pin, with a screw thread cut on the end to pass through the impression cup, to a shoulder, leaving a space of from one-eighth to one-half inch between the inside surface of the cup and plate, (this to be regulated according to the depth of arch in each particular case), a small nut is screwed on to the end, outside of the cup, which holds it firmly in its place; the cup is filled with plaster mixed with water in a semi-fluid state, passing under, around, and over the plate; when the plaster sets, the plate is firmly imbedded in the plaster; and in removing the cup from the mouth, all must come out together, retaining the plaster impression, in one solid piece. Without this contrivance, the plaster will frequently let go of the cup, and can only be removed in pieces.

We expect to have more to say on this subject in our next, and hope then to be able to explain it more clearly than time or space will permit at present.

### NEW DENTAL LATHE.

In the previous number of the Reporter, we advertised a "New Dental Lathe," with illustrative cut, which we have been manufacturing and selling with great satisfaction. In the present number will be found a new cut and description, illustrating a recent *improvement*, which makes the lathe still more desirable, and we anticipate a largely increased demand, especially as the price is not advanced with the improvement. See advertisements.

We found this lathe advertised in the *Dental News Letter*, WITHOUT A NAME. We procured one, and, upon close examination, failed to discover any name, mark, or sign, to indicate the inventor or manufacturer, from whence it came, or where it originated. With this state of facts, we went to work, got up patterns, and commenced manufacturing, and have continued to do so. Recently we have received satisfactory information to the effect, that HENRY SNOWDEN, of Baltimore, is the inventor and original manufacturer, and we take pleasure in thus giving him *credit* for the benefits which his *ingenuity* and *skill* have conferred upon the profession, and would be pleased if we could, in a more tangible form, express our appreciation and gratitude. Although without a personal acquaintance with Mr. Snowden, from what we know and have heard we can most cordially commend him to the confidence and patronage of the profession, but we would be doing the profession in the West, and ourselves injustice did we not avail ourselves of the advantages of this great manufacturing city, to manufacture here and supply the Dentists with goods of the best quality, at the lowest prices at which they can be obtained.

We are aware, that there are those who think that the sun rises and sets somewhere near New York, Boston, Philadelphia, and Baltimore; but we, of the Great West, must be permitted to believe otherwise, and the facts are apparent to every man who has *eyes and will see.*

In machinery, furniture, and clothing, Cincinnati has facilities equalled by few cities in the United States, surpassed by none. In materials, ingenuity, and skill, we have all the advantages, to make the best work, at the least expense. It would, therefore, be folly to get goods from the East, at a greater cost.

From the Scientific Artisan.

### THOUGHTS FOR THINKERS.

Sound travels at the rate of 1,142 feet per second in the air; 4,960 in water; 11,000 in cast iron; 17,000 in steel; 18,000 in glass, and from 4,636 to 17,000 in wood.

Mercury freezes at 38° Fahrenheit, and becomes a solid mass, malleable under the hammer.

The greatest hight at which visible clouds ever exist does not exceed ten miles.

Air is about 816 times lighter than common water.

The pressure of the atmosphere upon every square foot of the earth amounts to 6,160 lbs. An ordinary sized man, supposing his surface to be 14 square feet, sustains the enormous pressure of 30,340 lbs.

Heat rarifies to such an extent that it can be made to occupy 5,500 times the space it did before.

The violence of the expansion of water when freezing is sufficient to cleave a globe of copper of such thickness as to require a force of 28,000 lbs. to produce a like effect.

During the conversion of ice into water, 140 degrees of heat are absorbed.

Water when converted into steam, increases in bulk 18,000 times.

One hundred pounds of Dead Sea water contains 46 lbs. of salt.

The mean annual depth of rain that falls at the equator is 96 inches.

Assuming the temperature of the interior of the earth to increase uniformly at the rate of 1° for every 45 feet, at the depth of 60 miles the degree of heat would be sufficient to fuse all known substances.

The explosive force of close confined gun powder is six and a half tons to the square inch.

The greatest artificial cold ever produced is 91° Fahrenheit.

### ALUMINIUM.

It has been proposed by Mr. Corbelli, of Florence, Italy, to obtain aluminium by a simpler and more economical process than heretofore practiced, by employing the following process: The *metal is first well washed and cleansed from extraneous matters*, then he takes 100 grammes of *clay*, dries it, dissolves it in six times its weight of sulphuric acid, or very strong muriatic acid, dries the clay again and heats it in an earthen vessel to 450° or 500° C., after which he mixes with it 200 grammes of *yellow* prussiate of potash, which should be quite dry and pulverized. To this mixture he adds 150 grammes of common salt, and places the whole, when intimately mixed, in a crucible. He then heats them to a white heat, and after the mass is cool, the *aluminium* will be found at the *bottom* of the crucible.

We copy the above from the *Scientific Artisan;* the *Italics* are ours.—Eds.

### ANATOMICAL SPECIMENS.

We have for our own use, and also for sale, a few Specimens of 1st and 2d Dentition, as illustrated in the first part of *Reporter*; also, some beautiful specimens of Adult Jaws with perfect set of teeth; nervous system exhibited on one side and veinous and arterial on the other. Price $15 and $25.

### INDIANA STATE CONVENTION.

The Dentists of the State are invited to attend a Dental Convention to be held at Indianapolis on the 28th day of December next, for the purpose of forming a State Dental Convention. A circular of invitation will be sent to all practicing Dentists, whose names and residence can be obtained. All competent practitioners are cordially invited and earnestly solicited to attend.

Go in gentlemen. You can not engage in a better cause; the time, labor and money spent in such a cause is well invested and will pay an hundred fold, although it may not appear, it is even so, and to prove it we have only to point out the eminently successful, liberal minded, whole-souled, go-ahead and self-sacraficing professional men, every where! The example of the Hoosier State is worthy of emulation.

"Pitch in," "Buckeye Boys!" Let not OHIO be behind in any thing that promises advancement in science, literature and art! We hope ere long to see local societies in every large town, or in every District, and State societies in every State. It is this that gives tone and importance to the profession.

DR. ELISHA TOWNSEND, *of Philadelphia.*—The death of this worthy gentleman will give pain to a large circle of friends, and by his death science has lost a most intelligent servant. He was President of the Dental College, Philadelphia, and has been instrumental in doing much to advance the true interests of the profession throughout the country.

As a man he possessed noble qualities, and as a friend he was prompt, unselfish and sincere. He had recently visited Europe for his health, but returned only to die. He will long be remembered by those who knew him, and the absence of his aid and counsel will make a void not easily filled.

BOSTON, Oct. 18, 1858. E. G. T.

The above we find in the *Boston Medical and Surgical Journal*, and it has rarely fallen to our lot to witness so much astonishment, consternation and regret, as this announcement has produced among members of the Dental profession. All unite in offering to him that tribute of admiration and respect which is due to genius and devotion to science, for the benefit of his fellow men and the elevation of the profession which he practiced and loved. "*May he rest in peace.*"

### NEW PATENT—DENTAL CHAIRS.

ALEX. M. HOLMES, of Morrisville, N. Y.

I claim first, the foot Rest O, arranged with the Slides j j. Backs n, Pinions m, and palls *o*, substantially as described.

2d. The supplemental back P, attached to links g, which are fitted in the slot p of the back c and actuated by the set screw s, substantially as set forth.

3d. The adjustable head rest, formed of the slide u, pinion w, plates Q R and a'. b'. arranged relatively to each other and applied to the back c, substantially as set forth.

### NEW LAMP.

In the *Scientific Artisan*, we observe some cuts illustrating a new Lamp, to be applied to a variety of purposes,—cooking, bathing, melting metals, soldering, and in fact every thing for which a Dentist, Jeweler or a family would use a fire.

We have witnessed some experiments during the exhibition of the late fair of the Ohio Mechanic's Institute, and must confess to the conviction that for melting zinc, lead, or gold and silver, it takes the lead of any thing we have seen, both in economy of fuel and time; whilst in soldering it is equal to the best self-acting blow-pipes. We expect to see more and say more after a while.

### CHEAP BAROMETER.

Dissolve some camphor in alcohol and throw into the solution some soda. The camphor precipitates in snowy flakes, which are collected by passing the mixture through a filter, they are then collected and put into a vial containing a saturated solution of camphor (in strong alcohol.) The vial is then tightly corked and placed where it will not be disturbed, when it will prove an unerring index of the weather. In fine weather the precipitate rests on the bottom, but on the approach of a storm it will rise to the surface with a tendency to the quarter opposite to that from which the storm is coming, the flakes being effected electrically.

### CHEAP BAROMETERS.

Take a clean glass bottle and put in it a small quantity of finely pulverized alum, fill the bottle with spirits of wine, which will disolve the alum, and in clear weather the liquid will be clear and transparent, as pure water.

On the approach of rain or cloudy weather the alum will be visible in a flaky spiral cloud in the centre of the fluid, reaching from the bottom to the surface. This is a cheap, simple and beautiful barometer, and within the reach of all who wish to possess one.

### RILEY'S PORTABLE HAND LATHE.

This is a new Lathe, invented and constructed by Dr. W. W. Riley, of Columbus, O., who has kindly furnished us with the patterns, models and full instructions, for the manufacture of the article in its most perfect form. We give below a cut, illustrating it as it would appear on the bench in operation.

It runs with a *band* smooth and light, without noise and but little friction, giving great speed and power; the sliding rod admits of tightening the band as it becomes relaxed. It can be all taken apart and packed in a very small compass, to carry to any place in the country. It has also a water box with faucet to keep the stones constantly wet. Price of Lathe and Water Box, $10.

We expect to construct a wheel with supports to fasten to the floor any where, then by putting a small pully-wheel in place of the crank, it can be driven with the foot. The extra cost of this arrangement will be from $5 to $8.

### ALUMINIUM IN SURGERY.

A correspondent of the London *Lancet* recommends Aluminium Sutures as a substitute for silver in the union of wounds by first intention. It is cheaper, (less than half the price,) more pliable, does not blacken from contact with pus, and can now be obtained in Europe without difficulty.

We believe it has not yet been introduced in this country, but its great utility in various ways, both for domestic and scientific purposes, will doubtless make it abundant here.—*Boston Med. and Surg. Journal.*

---

ALUMINIUM has been introduced into this country and some few experiments made with a view to applying it to the various arts and sciences, and especially to DENTISTRY, for which its resistance of *acids* and extreme *lightness* would especially recommend it, but thus far the difficulty seems to be in finding a suitable *Solder*, on account of the low degree of heat at which it melts, it can be hammered, rolled and struck up into plates, but there is some mistake about the *price*, unless it is computed by volume instead of weight. One ounce of aluminium will occupy about as much space as 10 ounces of gold. We have a small quantity for sale to those who wish to experiment, at $10 per ounce.

---

### NEW AMALGAM.

We have it in its PURITY, manufactured at head quarters. Price $4 per oz.

Also, what is called "Townsend's Amalgam" being made by the formula of "New Amalgam," but commercial metals being used instead of those of ABSOLUTE PURITY, besides being combined with less care. Price $2 per oz.

---

### DR. ALLPORT'S DENTAL LEDGER.

This is a matter that, from the press of other business, has been neglected; we have intended for several months past to have some printed, and will certainly do so soon. All who wish to be supplied will please send in their orders in advance, so that we can judge of how large an edition is required.

### LUTHER'S FLASK.

For simplicity, convenience, *rapidity* and ACCURACY, this little arrangement has no equal, the better to accommodate the increased demand, we have had a larger size made so that the *largest* cases can be taken with facility. It needs no description, but requires only to be seen to be appreciated and adopted.

There are now *two* sizes. Price $1 each,

### PRIZE ESSAYS.

In previous issues we have detailed the merits of this production, if Dentists would have them freely circulated in their respective districts, much good might be effected, in giving to the public a just appreciation of the importance of Dentistry and, the value of operations properly performed.

We have some on hand to supply those in need.

Price, $6 per hundred; $1 per dozen; 10 cts. each.

### CORRECTION.

In the article on "INFLAMMATION," by C. C. Dills, in the last number of the *Reporter*, the definition, as given by us, differs so fatally from that of the *manuscript*, that we feel it our duty to make the correction, by giving, *in full*, the definition as we find it in the manuscript, viz:

"Inflammation may be defined to be a deviation from the healthy physiological condition of a part, *accompanied* by a perverted condition of the blood *and* blood vessels, and attended with pain, heat, redness, and swelling: with the action of the part partly increased *and* partly diminished; accompanied by general febrile action."

We must also acknowledge some typographical errors in the same article, which, however, we doubt not, the reader has had the good sense to charge to the right account.

Dr. D. is a young man, of tender feelings, and much promise, but a little too confiding and dependent on the opinions of others, which will, if not corrected, place him in many unpleasant positions; but experience and contact with the world will soon correct these faults, if faults they can be called.

Ohio College of Dental Surgery.—The session of 1858-9 commenced on Monday, the 1st day of Nov. Already we see a goodly number of students. The Professors are on hand with *great stores* of knowledge, which they are full of zeal to impart to the attentive class. We hope to see the Lecture Room and Labaratory crowded this winter.

Hail-stones sometimes fall with a velocity of 113 feet in a second, and rain at 43 feet in a second.

Lightning can be seen, by reflection, at the distance of two hundred miles.

The Francis' Patent Sustained by Law.—We are in receipt of a letter from James J. Clark, assignee and patentee of Francis' Electro-Magnetic process of extracting teeth without pain, in which he remarks, "we have just concluded our *Law Suit*, and have come out Victorious!"

The claim is to the "combination of the Electro-Magnetic machine with the Dental Forcep." The foot-key is covered in a separate patent.

Full office right including apparatus and instructions, $100.

Right to use the invention *six months* $30, with privilege to purchase the right for the remainder of the time for which the patents are issued for $70 more.

We are supplied with blank rights, and instruments with which to supply all who wish to adopt the process.

For particulars see advertisement in the *Reporter*. J. T. T.

The Mac-a-check Press.—Vol. 1. No. 1. reached us just in time to say a hurried word. In its typography it is the most beautiful specimen we have seen; clear, clean, and distinct, we pick it up to lay it down again, to fully assure ourselves that it is really only a country newspaper, it is so unlike and so much superior to others of like origin; the publishers must have read Shakspeare with a practical intent, as they seem to have adopted to the letter his advice,

"Costly thy habit as thy purse can buy,
But not expressed in fancy; rich, not guady;
For the apparel oft proclaims the man."

Besides the information in its reading &c., its advertising will interest and prove beneficial to all classes, and here let us remark, that whoever subscribes will be sure to be satisfied, and whoever advertises can depend on it he will get his money back.

In politics the *Press* proposes to support the only *live* organization which proposes "*fair and just* EQUALITY of *political rights*."

God speed you friend, since you have removed the *beam* from your own eye, you may see clearly the *mote* in thy brother's.

---

ELECTRICAL ANASTHESIA BY W. G. OLIVER.—This is the title of a pamphlet of 28 pages, issued by Dr. W. G. Oliver, of Buffalo, N. Y., it comprises a brief history of the discovery, and a synopsis of experiments, also full directions for its application in *Surgical* and *Dental operations*. Dr. O. remarks in reference to extracting teeth, "with the Magneto-Electric machine our successful cases averaged about 60 per cent. whereas with the vibratory machine our success was full 90 per cent. and now after a years experience it is *certainly* 98 *per cent.* besides having devised means for applying the battery to prevent pain, in every other operation appertaining to the Dental profession," in another part of the work the author remarks, "the question has frequently been asked, will Oliver's process infringe upon the Francis' patent! in my opinion, decidedly NO, *they are very different*, and as to the question of superiority the profession must decide."

The price of the book is $1, and can be obtained direct from the author, or will be supplied by your humble servant, J. T. T.

☞ See advertisement.

---

THE URÆMIC CONVULSIONS OF PREGNANCY, PARTURITION, AND CHILDBED, BY DR. CARL R. BRAUN, Professor of Midwifery, Vienna. Translated from the German, with notes by J. Matthews Duncan, F. R. C. P. E.—This is a Book of 182 pages, elegantly bound, and printed on good white paper. It is published by S. S. & W. Wood, 389 Broadway, N. Y. Price 75 cents, (free of postage,) and for sale in Cincinnati by the enterprising firm of Rickey, Mallory & Co.

It is an able, careful, and learned treatment of the subject, and brings before the American reader the observations and experience of one of the best of German Physicians; his exposition of a subject which is one of the most prominent in the whole range of practical medicine, and the utter deficiency of any similar treatise in the English Language, makes it more valuable and desirable.

Students in every branch of medicine are aware of the deficiency of laborers in the field of organic chemistry of human Pathology, probably the most important branch of that great science. Dr. Braun's work shows the advantages derived from a mere intimate alliance between such scientific studies and the pursuits of practical physicians. No physician or student should be without this book.

---

MERRY'S DRILL STOCK.—In another part of this No. (page 125,) will be found a cut and description of this very ingenious instrument.

We are now manufacturing them for sale. Price of Drill Stock with one doz. Drills, $4, or $3 for the Stock and $1 per doz. for Drills.

---

FORBES' SOCKET HANDLE.—On page 124 of this No. of Reporter, will be found a cut and description of a Socket Handle introduced to the profession by Dr. Isaiah Forbes, of St. Louis. We are manufacturing them, and will be ready to furnish the profession in a few days, as follows:

| | | |
|---|---|---|
| Ivory Handled Socket, with 1 doz. Bits, | - - | $4 50 |
| Ebony " " " " " | - - | $3 50 |

## Figures and Tables

**Figure f1:**